# The British Medical Association at Bristol

**Published:** 1894-09-08

**Authors:** 


### THE BRITISH MEDICAL ASSOCIATION AT BRISTOL.

### ANNUAL MUSEUM.

(FOURTH NOTICE.)

HERTZ AND COLLINGWOOD, 4, Sussex Place, Leadenhall Street, showed the Laurent Perrier "Champagne sans Sucre," a wine of splendid flavour and bouquet. They provided a generous supply of this for the Conversazione, and its first-rate quality will be long remembered. It is employed in the manufacture of their Coca Tonic Champagne with the result of making a most delicious restorative. They showed also the Rosbach Table Water; the Levico, an arsenious water; and the Franz Joseph, a palatable purgative water.

THE JEYES' SANITARY COMPOUNDS COMPANY were as usual strongly represented, especially in those varieties of their products which are particularly adapted for surgeons. Thus they showed Creolin gauze; Creolin powder, a substitute for iodoform, and valuable in domestic use for perspiration of the feet; Creolin ointment; and Creolin itself, the well-known non-poisonous disinfectant. A new article of interest also was to be found in their "Symphoral," a compound of caffeine and sulphonic acid, which has been highly recommended as a diuretic without causing unpleasant after-effects.

BURROUGHS, WELCOME, AND CO. appeared in two parts of the hall. They had a beautifully decorated stall at the entrance, where they offered refreshments to visitors in the form of tea prepared from their new tabloids. This was a great centre of attraction, and the wonderful ease with which a cup of good tea can be made in a few moments excited general admiration. The method of making tea into tabloids has long been employed in Japan, and has the advantage of obtaining much more strength from a given quantity of tea that the ordinary infusion of loose leaves does; but Messrs. Borroughs and Welcome have introduced considerable improvements, and the new tabloids are certain to have a very great demand. The tabloids are by far the most economical, quick, and easy method of making tea. In their other stall we noticed the usual artistic arrangement of their multitudinous articles. Their Emol Keleet, which was described at Newcastle last year, is a dusting powder, with many advantages over ordinary Fuller's earth, for eczema and other skin affections. Their ophthalmic tabloids (and cases) contain dilating and other agents in a solid form to be placed in the eye. They are at once dissolved by the lachrymal secretion, and save the surgeon from carrying drop-bottles about with him. The photographic tabloids have the special advantages of affording an accurate measure and stable reagents, which photographers will fully understand the value of. Their medicine chests for travellers and home use are full of every improvement, and one of them has just been sent out in the Harmsworth Arctic Expedition. The powder insufflator is both light and convenient to use, and has separate containers for different powders. The soloids for making antiseptic solutions are too well known to need description, and the endless catalogue of their exhibits precludes more than a passing mention. The Lawton Absorbent Cotton, the antibilious cocoa, the Hazeline, and Kepler products are worth remembering.

S. KUTNOW AND CO., Holborn Viaduct, pointed out the cheapness and portability of their effervescent Carlsbad sprudel salts, dried and taken from the waters of the actual spring, and specially purified. Another important exhibit was their anti-asthmatic cigarettes and powder, containing neither lobelia nor datura tatula, and hence less depressing to the patient. They may be used not only for asthma, but also for ordinary colds and bronchitis. A small teaspoonful of the powder burned on a plate is said to give very rapid relief.

BOYD AND CO., Belfast, attracted much attention by their exhibit of the doctor's Ulster, with pockets for instruments, the mackintosh, the golf cloaks for ladies, and their beautiful eiderdown rugs. The lightness and warmth of these articles was astonishing, and makes them very acceptable as presents to invalids and to those who have to face severe weather.

G. MELLIN showed many modifications of his celebrated foods. Thus the Lacto-Glycose is a mixture of Mellin's food and pure milk, and the food biscuits are a palatable dry form, to which wheat flour has been added. The emulsion of oil and hypophosphites is well made, and the emulsion has been carried out without the addition of an alkali. Besides this it is readily miscible with water, milk, and other fluids. The unsweetened coca wine, prepared with a simple red wine, is another palatable preparation.

Van Abbott and Sons, Grosvenor Square, offered a selection of their new preparations for diabetics. These are a great improvement on the old tasteless foods, and will be a wonderful boon to those who have to cater for such patients. The biscuits and confectionery made without sugar, the chocolate, the almond flour, the soya and gluten bread, the bran powder, and the macaroni afford a surprising variety of foods. Nor should we forget their hypophosphite of lime biscuits, which have been recommended and analysed by the *Lancet*. Each contains five grains of hypophosphites.

The Flitwick Chalybeate Company showed their powerful water from the Bedfordshire spring. We find by experiment that it is very readily taken, and certainly the analysis shows an extraordinary amount of iron in the ferric state held in solution. A special advantage claimed for it is that it does not discolour the teeth or produce constipation. For anæmia, chlorosis, &c., it has been already reported upon most favourably. Their beautiful advertisement in the shape of a photogravure of Gainsborough's "Cottage Girl" is a striking success.

The "Viking" Food and Essence Company's preparations were tasted by great numbers. Their essences of beef, mutton, chicken, and turtle are best taken cold, and their turtle soup and jelly, and their pure solid beef tea are very delicious. They had also their concentrated meat juice, beef jelly, the "South American" brand of Liebig' Extract, and the Viking sauces in great variety. Kopf's consolidated soups, pea,, lentil, and Scotch broth in the solid form, have been largely used by Government and are well known. The Idris table waters were supplied in immense quantities for the conversazione, and greatly appreciated.

The Sanitas Company showed an enormous number of the Sanitas products, the result of Mr. Kingzett's researches. The Sanitas fluid, oil, inhalers, soaps for men and animals, the eucalyptus disinfectors and oil, and a great variety of toilet preparations, such as Sanitas cream and tooth powder, were among the most interesting articles. In short, "Nature's only disinfectant," derived from pine, eucalyptus, and camphor forests, is presented in endless forms. The Anti-diphtherite is an apparatus of the respirator type for supplying disinfected air to persons engaged in attending upon diphtheritic and other patients. Many a life might be saved by its use among nurses and medical men.

F. Canton and Co. were noticeable for their pure grape brandies. The *Lancet* reported that its analysis of them showed an "entire absence of the raw products which are the accompaniment of spirit produced from potatoes and cereals," besides other indications of the origin of these alcohols from the grape. The aroma and flavour are of high character, and, indeed, these brandies appear well fitted for medicinal use.

Parke, Davis, and Co. had a large assortment of drugs presented in standardised form by very palatable methods. Mosquera's beef jelly is a delicious meat food, and can be had either in a solid or liquid form. One great speciality of this firm is the number of American and foreign drugs which they introduce to the English market. Coquillaña, as a remedy for bronchitis, the Hydrastis Canadiensis for dyspepsia and chronic inflammations of mucous surfaces, and many others were shown by them. Their aluminium hypodermic case is a beautiful little contrivance, and their capsules, pills, and tablets showed great excellence in the manufacture.

The Cerebos Salt Company exhibited samples of their table salt, which contains the mixed phosphates of bran. It is a beautiful salt, fine in grain, and does not cake when in use. It can be employed as a daily article of diet, or in the manufacture of ordinary bread, with the view of supplying those elements which are wanting in the commonly used white flour.

Krohne and Sesemann.—Their improved inhalers for use in giving anæsthetics are of the highest importance, insuring accurate measurement and control of the vapour given and the due supply of air. The consumption of the anæsthetic is made more economical, as well as far safer. Two new instruments devised by Dr. J. Mackenzie were shown, the one for taking tracings of the venous pulse, and the other—the polygraph—for combined tracings of the radial, the venous, and liver pulses, or the respiratory movements in general practice by the bedside.

Oliver's cephalograph for obtaining tracings of the pulsation of the brain, and their many new instruments in laryngology, nasal and aural surgery, were well worthy of careful attention. Dr. Harrison's new lithoclasts and perineal evacuating catheters were flanked by Mr. Fenwick's caisson tubes and forceps for bladder operations and the instruments used at the Samaritan Hospital for Women. We must not forget Schnid's self-suspension apparatus for home use in preventing and curing lateral curvature. Their exhibition altogether was a most instructive and interesting one to the modern surgeon.

